# 16S rRNA amplicon sequencing of organic and conventional chickens

**DOI:** 10.1128/mra.01081-24

**Published:** 2025-01-17

**Authors:** Anuradha J. Punchihewage-Don, Nur A. Hasan, Salina Parveen

**Affiliations:** 1 Food Microbiology and Safety Laboratory, Department of Agriculture, Food and Resource Sciences, University of Maryland Eastern Shore, Princess Anne, Maryland, USA; 2 EzBiome Inc., Gaithersburg, Maryland, USA; The University of Arizona, Tucson, Arizona, USA

**Keywords:** 16S rRNA, organic, conventional, chicken, sequencing, amplicon, microbiomes, whole-carcass enrichment, whole-carcass rinse, V3-V4 region

## Abstract

We report 16S rRNA sequencing of microbiomes associated with organic and conventional retail chickens (*n*=31), processed via whole-carcass enrichment and rinse methodologies. Amplicon sequencing was performed targeting the V3-V4 region of the 16S rRNA gene, which resulted with Good’s coverage exceeding 99.7% of the libraries.

## ANNOUNCEMENT

Chicken, a popular meat in the U.S.A., is often contaminated with foodborne pathogens like *Salmonella* (1–3). The United States Department of Agriculture Food Safety and Inspection Service recommends frequent *Salmonella* testing using whole-carcass enrichment (WCE) and whole-carcass rinse (WCR) methods (4–6). This study evaluated the effectiveness of these methods in detecting pathogens in organic and conventional retail chickens using 16S rRNA sequencing (7).

Chicken carcasses (*n* = 31) were collected from a Maryland retail store (38.420904° N, 75.563683° W) and processed as described in our previous study (7). Each carcass was mixed with 500 mL buffered peptone water (BPW), and 30 mL of the resulting liquid was combined with 30 mL fresh BPW for the WCR method. The remaining liquid and carcass were used for the WCE method. Both samples were incubated at 37°C for 24 hours. Fifty milliliters of incubated samples from WCE and WCR was divided into four groups: organic rinsed (*n* = 7), organic enriched (*n* = 8), conventional rinsed (*n* = 7), and conventional enriched (*n* = 9). A negative control (BPW only, *n* = 5) was included. Samples were filtered through 1.0 and 0.22 micron filters using a mechanical pump and Sterivex unit. DNA was extracted from the filters using the DNeasy PowerWater Kit, per the manufacturer’s instructions.

The 16S rRNA gene V3-V4 region (341–805 nt) was sequenced for all samples using the paired-end Illumina MiSeq platform. A two-step PCR was employed. In the first step, each 25 µL reaction included 2.5 µL of 1× primer mix, 5–50 ng metagenomic DNA, 0.5 µL AccuPrime Taq DNA Polymerase, and 2.5 µL of 10× AccuPrime PCR Buffer II (Thermo Fisher). PCR conditions included an initial denaturation at 95°C for 2 min, followed by 10 cycles at 95°C for 45 s, 57°C for 90 s, and 72°C for 50 s, with a final extension at 72°C for 10 min. The resulting PCR products were pooled and used to create Illumina dual-index libraries with 8 bp indexes for multiplexing. Each reaction contained 0.5 µL AccuPrime Taq DNA polymerase, 2.5 µL 10× AccuPrime PCR buffer II (Thermo Fisher), 3 µL each of D50x and D70x adapter primers, 4 µL of 10 µM Illumina primers, and 50 µL PCR product from the first reaction. PCR conditions included denaturation at 95°C for 2 min, followed by six cycles at 95°C for 45 s, 60°C for 30 s, and 72°C for 50 s, with a final extension at 72°C for 10 min. Dual-indexed libraries were purified using Ampure beads (Beckman Coulter), quantified with Qubit dsDNA HS assay (Thermo Fisher), and validated on a Bioanalyzer (Agilent). Final quantitative PCR quantification was performed before loading onto a MiSeq sequencer for PE250bp (v.2 chemistry).

**Fig 1:**
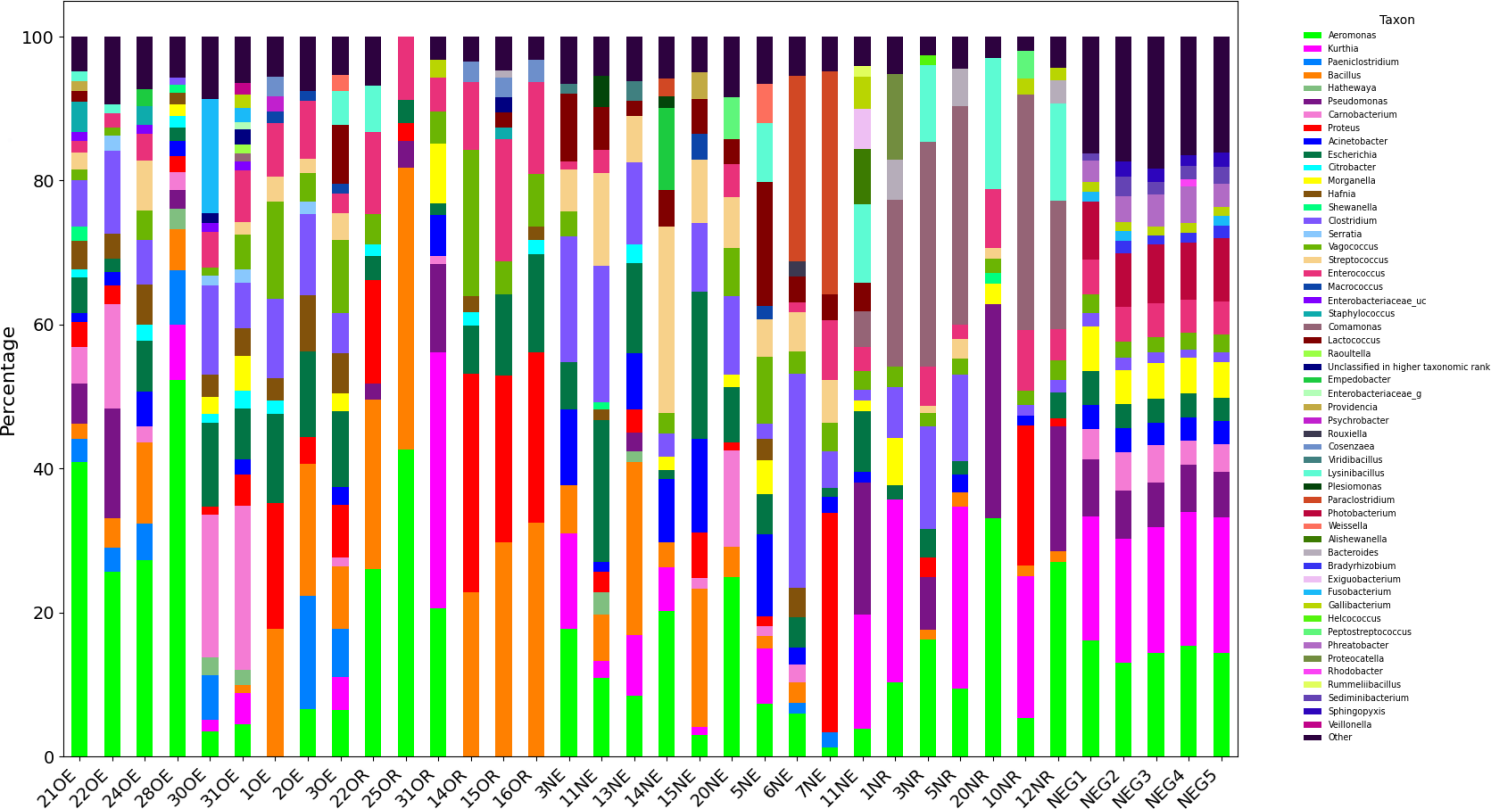
Relative abundance of microbial genera for each chicken sample (1% cutoff applied; genera with relative abundances below 1% were aggregated into an “other” category). The plot was generated using Python (v.3.12) with Matplotlib for visualization and distinctipy for distinct color combinations.

Taxonomic profiling was conducted on the EzBioCloud platform (v.PKSSU4.0) (8) with default parameters, yielding high-quality reads averaging 79,184,727 bases, a GC content of 53.2%, and file sizes averaging 43.92 MB (Table 1). The Illumina read length was 251 bp. Good’s coverage exceeded 99.7% across all cohorts. Overall, the bacterial community was diverse, consisting of 11 phyla, 25 classes, 55 orders, 101 families, 243 genera, and 752 species, including unclassified taxa. Figure 1 shows the relative abundance of microbial genera across chicken samples.

**TABLE 1:** BioSample information for 16S rRNA sequencing of microbiomes from organic and conventional retail chickens

SRA run accession	Sample ID	GC%	Bases	Number of reads	Treatment
SRR26222301	15OR	53.1	76,134,324	303,324	Organic rinsed
SRR26222302	14OR	53.0	65,087,312	258,495	Organic rinsed
SRR26222303	31OE	53.2	83,096,562	331,062	Organic enriched
SRR26222304	28OE	53.9	123,773,622	493,122	Organic enriched
SRR26222305	24OE	53.7	90,322,350	359,850	Organic enriched
SRR26222306	22OE	53.34	87,889,156	350,156	Organic enriched
SRR26222307	NEG5	53.2	94,436,240	376,240	Negative control
SRR26222308	NEG4	53.3	79,931,954	318,454	Negative control
SRR26222309	NEG3	53.1	69,736,334	277,834	Negative control
SRR26222310	NEG2	53.2	64,425,174	256,674	Negative control
SRR26222311	NEG1	53.3	75,821,578	302,078	Negative control
SRR26222312	20NR	53.3	68,240,876	271,876	Conventional rinsed
SRR26222313	21OE	53.8	80,588,068	321,068	Organic enriched
SRR26222314	5NR	53.0	64,892,034	258,534	Conventional rinsed
SRR26222315	3NR	53.7	70,363,332	280,332	Conventional rinsed
SRR26222316	1NR	53.0	85,133,678	339,178	Conventional rinsed
SRR26222317	11NR	53.4	61,676,724	245,724	Conventional rinsed
SRR26222318	12NR	53.7	62,003,024	247,024	Conventional rinsed
SRR26222319	10NR	53.2	69,138,452	275,452	Conventional rinsed
SRR26222320	20NE	53.5	75,908,424	302,424	Conventional enriched
SRR26222321	15NE	53.0	74,114,276	295,276	Conventional enriched
SRR26222322	14NE	52.7	90,025,166	358,666	Conventional enriched
SRR26222323	13NE	53.1	80,272,310	319,810	Conventional enriched
SRR26222324	3OE	53.1	152,608,502	608,002	Organic enriched
SRR26222325	11NE	53.3	80,486,664	320,664	Conventional enriched
SRR26222326	3NE	52.9	71,607,790	285,290	Conventional enriched
SRR26222327	7NE	52.3	63,852,392	254,392	Conventional enriched
SRR26222328	6NE	52.5	68,509,446	272,946	Conventional enriched
SRR26222329	5NE	52.5	73,042,506	291,006	Conventional enriched
SRR26222330	31OR	53.0	73,360,774	292,274	Organic rinsed
SRR26222331	30OR	53.0	76,119,264	303,264	Organic rinsed
SRR26222332	25OR	53.7	76,665,440	305,440	Organic rinsed
SRR26222333	22OR	53.5	87,112,060	347,060	Organic rinsed
SRR26222334	16OR	53.2	75,867,260	302,260	Organic rinsed
SRR26222335	2OE	53.5	75,696,580	301,580	Organic enriched
SRR26222336	1OE	53.1	82,710,524	329,524	Organic enriched

## Data Availability

The 16S rRNA sequencing data generated for this study have been deposited in the National Center for Biotechnology Information Sequence Read Archive under BioProject accession number PRJNA1021594. The individual accession numbers (SRR26222301–SRR26222336) are included in Table 1. The raw reads are publicly available through this BioProject.
